# Patterns of Urinary Neutrophil Gelatinase-Associated Lipocalin and Acute Kidney Injury in Neonates Receiving Cardiopulmonary Bypass

**DOI:** 10.3390/children7090132

**Published:** 2020-09-09

**Authors:** Kathleen G. Brennan, Elvira Parravicini, John M. Lorenz, David A. Bateman

**Affiliations:** Department of Pediatrics, Vagelos College of Physicians and Surgeons, Columbia University Medical Center, New York, NY 10025, USA; ep127@cumc.columbia.edu (E.P.); jl1084@columbia.edu (J.M.L.); dab2@cumc.columbia.edu (D.A.B.)

**Keywords:** acute kidney injury, neonates, urinary neutrophil gelatinase-associated lipocalin (uNGAL), cardiopulmonary bypass

## Abstract

Elevated urinary neutrophil gelatinase-associated lipocalin (uNGAL) predicts acute kidney injury (AKI) in children following cardiopulmonary bypass (CPB) during cardiac surgery, but little is known about uNGAL’s predictive ability in neonates in this setting. We sought to determine the relationship between AKI and post-CPB uNGAL in neonates in the first 72 post-operative hours. Methods: Urine samples for uNGAL analysis were collected at preoperative baseline and serially post-operatively from 76 neonates undergoing CPB. Mixed-effects regression models and logistic models assessed associations between uNGAL and AKI (controlling for sex, gestational age, CPB time, surgical complexity, and age at surgery). Receiver-operator curves were applied to define optimal uNGAL cut-off values for AKI diagnosis. Results: Between 0 and 4 h post-operatively, uNGAL values did not differ between neonates with and without AKI. After 4 h until 16 h post-operatively, significant time-wise separation occurred between uNGAL values of neonates with AKI and those without AKI. Odds ratios at each time point significantly exceeded unity, peaking at 10 h post-operatively (3.48 (1.58, 8.71)). Between 4 and 16 h post-operatively, uNGAL discriminated AKI from no-AKI, with a sensitivity of 0.63 (0.49, 0.75) and a specificity of 0.68 (0.62, 0.74) at a cut-off value of 100 ng/mL. Conclusion: After 4 h until 16 h post-operatively, elevated uNGAL is associated with AKI in neonates receiving CPB during cardiac surgery; however, this relationship is more complex than in older children.

## 1. Introduction

Acute kidney injury (AKI) is a well-established risk factor for morbidity and mortality in hospitalized patients [1,2], especially among neonates undergoing cardiac surgery who have a reported incidence of AKI up to 60% [3,4,5,6]. Even mild degrees of post-operative AKI can lead to significant increases in adverse outcomes, including longer hospital stays, longer ICU times, and diminished quality of life [7,8,9].

AKI definition continues to evolve, with more than 30 definitions of AKI present in published literature [1,4]. Increasingly, the pediatric and adult communities have adopted the Kidney Disease: Improving Global Outcomes (KDIGO) criteria as the predominant definition for AKI [10]. KDIGO criteria, like prior definitions of AKI, are based on varying degrees of change in serum creatinine (sCr) levels and urine output—delayed consequences of kidney injury rather than direct markers of injury. In neonates, using sCr and urine output to define AKI is further complicated by the unique characteristics of neonatal physiology and the impact of maternal renal function [3,11,12]. In the first weeks of life, sCr levels predominantly reflect maternal renal function and change rapidly, making accurate calculation of glomerular filtration rate impossible as sCR is not in a steady state. Additionally, sCr levels are substantially influenced by weight, muscle mass, and serum bilirubin levels in these vulnerable neonates. Consequently, evaluation of neonatal renal function and diagnosis of neonatal AKI are particularly challenging. Interest in improving the diagnosis of AKI has led to the investigation of a number of renal injury biomarkers. Among these biomarkers, urinary neutrophil gelatinase-associated lipocalin (uNGAL) is perhaps the best studied in older children and adults. In these older children and adults, uNGAL is well correlated with and is an early predictor of AKI in a variety of clinical settings such as after exposure to cardiopulmonary bypass (CPB) during cardiac surgery. However, despite the high rate of post-operative AKI in neonates undergoing cardiac surgery, there remains limited information about the correlation between uNGAL and AKI in these neonates [12,13,14,15]. Given their elevated risk for AKI and the unique neonatal confounders of current AKI definitions, an accurate biomarker for neonatal AKI could substantially change our ability to identify and, therefore, support vulnerable neonates.

The aim of this study was to investigate whether increases in uNGAL correlate with AKI in neonates following cardiopulmonary bypass. We hypothesized that, as with older children, uNGAL elevations would be associated with AKI in neonates undergoing cardiac repair using CPB.

## 2. Materials and Methods

### 2.1. Subjects and Study Design

**Figure 1:**
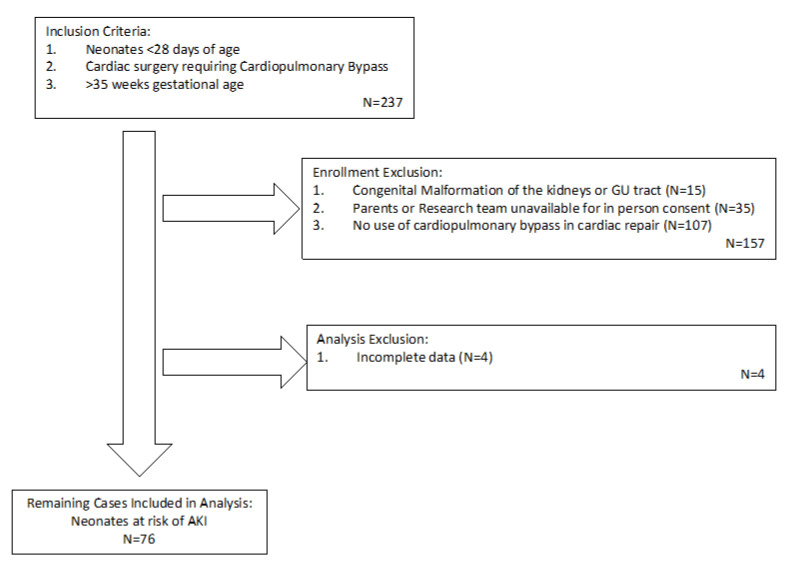
Flow chart of enrollment. AKI, acute kidney injury; GU, genitourinary.

In this prospective observational study, we consecutively enrolled neonates <28 days of age who required cardiopulmonary bypass (CPB) during cardiac surgery. All patients with congenital heart defects at our center, Columbia University Medical Center, over a two-year period were screened for study inclusion. Patients with known congenital malformations of the kidneys or genitourinary tract or who were <35 weeks gestational age (considered premature birth) were excluded. During the study period, 237 neonates were screened for participation in this study. Of these, 157 neonates were excluded because they had congenital cardiac disease that did not require use of cardiopulmonary bypass during surgical repair, there was a known congenital malformation of the kidneys, or because either research team or their parents were not available for in person consent prior to surgical repair. No families declined enrollment. Four patients were removed from analysis due to incomplete data, leaving 76 patients available for analysis (Figure 1). This study was approved by the Institutional Review Board of Columbia University Medical Center (study number AAAN8206) and followed the rules of the Declaration of Helsinki; written parental consent was obtained prior to each neonate’s inclusion in this study.

The primary outcome measure was presence of AKI. AKI was defined as neonatal KDIGO stage 1 or higher—increase in sCr (≥0.3 mg/dL or ≥ 50% increase from previous lowest value) or decreased urine output (<1.5 mL/kg/h for 24 h) [5,15]. In a prior retrospective review to determine the internal prevalence rate of neonatal AKI following cardiac surgery at our institution, all cases of AKI developed in the first 72 h post-operatively; for this reason, AKI was defined as neonatal KDIGO stage 1 or higher at any point in the first 72 h following cardiac surgery.

### 2.2. Protocol

Urine samples for biomarker analysis were obtained immediately preoperatively and then between >1 and ≤3 h post-operatively (labeled 3 h), >3 to ≤ 4 h post-operatively (labeled 4 h), and then every 3 h for the first 24 h post-operatively (labeled 7, 10, 13 and 16 h, etc.). Urine samples were collected with indwelling catheters, which were placed as part of standard-of-care perioperative treatment for patients undergoing cardiac surgery in our unit. Urine samples were stored in aliquots at −80 °C. uNGAL concentrations were then measured by the Biomarkers Lab at Columbia University using a commercially available ELISA kit (ENZO Life Sciences, Inc, Farmingdale, New York, NY, USA). Laboratory investigators were blinded to clinical outcomes. Results of uNGAL analyses were unknown to clinical investigators during the hospital admission.

The serum creatinine (sCr) was measured for clinical practice. The perioperative practice for these patients is to measure sCr at baseline (within 48 h prior to surgery) and at least daily in the post-operative period. 

In the 24 h preoperatively and the 72 h post-operatively, the following clinical parameters were recorded: time on bypass, aortic cross-clamp time, circulatory arrest time, antegrade cerebral perfusion time, diuretic use, pressor use (e.g., milrinone and epinephrine), and urine output. The Risk Adjustment for Congenital Heart Surgery (RACHS-1) was used to stratify the complexity of cardiac surgeries performed [16].

Prior to study onset, a perioperative protocol for fluid resuscitation and steroid use was implemented to standardize practice for neonates undergoing cardiac surgery requiring cardiopulmonary bypass; thus, all neonates included in our study received the same intra-operative steroid dose (methylprednisolone 2 mg/kg). There were no significant changes to practice or personnel during the study period. 

### 2.3. Power Analysis

Our previous analysis exploring the relationship between uNGAL values and AKI in very low birthweight infants demonstrated that uNGAL >50 ng/mL had a sensitivity of 66% and a specificity of 66% to predict AKI [14]. Using this data in combination with our institutional prevalence rate of 25% for post-operative AKI in neonates undergoing cardiac surgery with CPB, we performed a power analysis to determine the number of neonates required to discriminate sensitivity and 1-specificity. This analysis demonstrated that to obtain 80% power to detect significant discrimination with a probability >95%, 71 patients (18 with expected AKI, 53 without) were required. To account for possible dropout and incomplete data, study enrollment was continued until 80 patients were enrolled.

### 2.4. Statistical Analysis

Bivariate analyses used Student’s t, Mann–Whitney, chi-squared and Fisher exact tests, as appropriate, to assess associations between predictor variables and AKI. Results were considered statistically significant at *p* < 0.05. 

**Figure 2:**
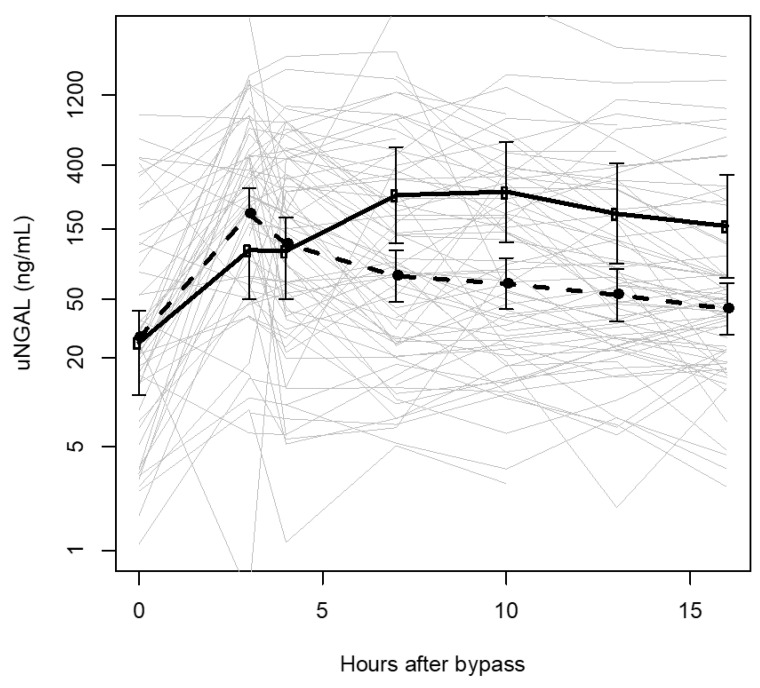
Mean uNGAL levels over time in neonates with and without AKI. Mean uNGAL concentrations (ng/mL) at collection times after cessation of cardiopulmonary bypass in patients with AKI (open circles, solid line) and without AKI (closed circles, dashed line). Mean log(uNGAL) values and error bars representing ± 95% CIs were derived from a hierarchical mixed-effects regression model of the interaction of AKI status and time. Values shown on the *y* axis scale have been exponentiated. Individual trajectories of log(uNGAL) are shown using thin gray lines.

Prior studies have shown that uNGAL has a log-normal distribution [17]. To generate Figure 2, mean log(uNGAL) values and 95% confidence intervals at each uNGAL collection point were derived from a single hierarchical mixed-effects model regressing log(uNGAL) on the interaction of AKI status and time interval, controlling for within-subject correlation, sex, gestational age (GA), CPB time, RACHS-1 score, and age at surgery. In logistic regression models at each collection interval, we used AKI as the dependent variable and log(uNGAL) as the predictor of interest, controlling for the same set of covariates. Terms were removed from models when the probability of chance association with outcome exceeded 0.1. We used a receiver-operator curve (ROC) to explore uNGAL cut-off values that would optimize discrimination of AKI from non-AKI status. Statistical analyses were conducted using R (R Core Team (2019). R: A language and environment for statistical computing. R Foundation for Statistical Computing, Vienna, Austria. URL https://www.R-project.org/).

## 3. Results

AKI within the first 72 post-operative hours occurred in 22% (17 of 76) of neonates (Table 1). This corresponded well to our expected prevalence rate of 25%. AKI was sustained (72 h or longer) in 12% (6 of 76) of neonates. Neonates were divided into two group—those who developed AKI and those who did not. Because of the small number of neonates with stages 2–3 AKI (Table 2), all stages of AKI were combined into a single binary variable (AKI or No AKI). Neonate characteristics, shown in Table 1, appear generally similar between those with and without AKI, with the exception that neonates who developed AKI were older at time of surgery than neonates without AKI (median 7 days vs. 5 days, *p* = 0.01). However, at the time of surgery, 90% (68 of 76) of all neonates studied were young (≤10 days of age). Bypass times were similar in neonates who went on to develop AKI and those without AKI, but both groups were exposed to prolonged CBP times (>120 min). No study neonates required renal replacement therapy (RRT) or died. 

Gestational age, cardiopulmonary bypass time, cross-clamp time, antegrade cerebral perfusion time, circulatory arrest time, and surgical complexity (RACHS) were not different between groups. Over 30 different congenital heart defects were observed. The most frequent cardiac lesions were Transposition of the Great Arteries (*n* = 21) and Hypoplastic Left Heart Syndrome (*n* = 18). Given the wide variety of cardiac lesions, numbers of each cardiac lesion were too small for individual analysis; instead, cardiac lesions were grouped by surgical complexity (RACHS). RACHS scores were not different between neonates with and without AKI. 

Neonates could meet KDIGO criteria through either increased creatinine or oliguria or both, as defined by neonatal KDIGO criteria [5]. Preoperative sCr was sampled at a median time of 15.9 [11.6–32.9] h before surgery. Median [IQR] baseline sCr value was 0.50 [0.38–0.64] mg/dl and did not differ by AKI status (Table 1). None of the neonates studied met criteria for AKI preoperatively. Post-operative sCr was first sampled at a median of 0.52 [0.40–0.61] h post-operatively. For 45% (34 of 76) of study neonates, immediate post-operative serum creatinine (sCr) values were lower than preoperative sCr values (Table 1). Neonates with AKI and those without AKI were equally likely to have post-operative sCr values lower than their preoperative baseline sCr (29% (5 of 17) vs. 49% (29 of 59), *p* = 0.15). An additional 14% (11 of 76) of neonates had post-operative sCr values that were equal to their preoperative values. Lower post-operative sCr was unrelated both to duration of bypass and to complexity of cardiac surgery (RACHS score). 

Figure 2 shows a plot of log(uNGAL) vs. the time interval of post-operative urine collection in hours. Individual uNGAL trajectories by subject, shown in light gray, demonstrate wide uNGAL variability even at baseline, where one-third of uNGAL values exceeded 50 ng/mL, the predictive cut off for AKI noted for older children and very low birthweight infants [14,18,19,20,21] (Table 1, Figure 2). Mean trajectories for those with and without AKI are derived from a single, mixed-effects hierarchical model regressing log(uNGAL) on the interaction of time interval and AKI status. Neonates both with and without AKI showed an early (<4 h post-operatively) rise in uNGAL, the magnitude and trajectory of which were indistinguishable. After 4 h until 16 h post-operatively, significant time-wise separation between mean ± 95% CI log(uNGAL) values for those with AKI and those without AKI was observed. After 16 h (not shown), significant separation was no longer observed. 

To test whether AKI was related to uNGAL at each collection interval, we computed odds ratios using logistic regression models controlling for sex, gestational age (GA), CPB time, RACHS-1 score, and age at surgery, with AKI as the outcome and log(uNGAL) as the predictor (Table 3). Odds ratios significantly exceeded unity at each interval after 4 h up to 16 h post-operatively, peaking at 10 h (3.48 (1.58, 8.71)). None of the other covariates was significantly associated with AKI in these models. Median values at each collection interval for those with and without AKI followed a similar pattern (Table 3).

We used receiver-operator curves (ROCs) to explore cut-off values for uNGAL that would optimize discrimination of AKI from non-AKI by uNGAL. A cut-off value of 50 ng/mL, recommended by Mishra et al. and others as predictive of AKI in older children [18,19,20,21], appeared too low as it was exceeded by nearly all of the median values for neonates without AKI (Table 3). At a cut-off value of 100 ng/mL, screening test parameter estimates had a sensitivity of 0.63 (0.49, 0.75), a specificity of 0.68 (0.62, 0.74), a positive predictive value of 0.34 (0.25, 0.45), a negative predictive value of 0.88 (0.81, 0.92) and a likelihood ratio of 1.99 (1.51, 2.63). At a cut off of 185 ng/mL, a cut off recommended by Krawczeski et al. from their analysis of a subset of 35 neonates [19], parameter estimates in our population had a sensitivity of 0.57 (0.44, 0.71), a specificity of 0.78 (0.72, 0.83), a positive predictive value of 0.40 (0.30, 0.52), a negative predictive value of 0.88 (0.82, 0.92) and a likelihood ratio of 2.65 (1.90, 3.69).

We used these two uNGAL cut offs (100 and 185 ng/mL) to assess the relative timing of uNGAL elevation and AKI onset. For neonates with AKI, uNGAL values exceeded 100 ng/mL in 82% (14 of 17) of cases, with a median [IQR] of 3.0 h [3,4] and a mean ± SD of 3.5 ± 2.4 h post-CPB. For neonates without AKI, uNGAL values exceeded 100 ng/mL in 73% (43 of 59) of cases, with a median [IQR] of 3.0 h [3]. Time of AKI diagnosis was made at a median [IQR] of 12.0 h [8.9,23.4] and a mean ± SD of 16.3 ± 10.9 h post-CPB. Elevation of uNGAL >100 ng/mL occurred at a median of 8.7 [6.0,16.4] and a mean ± SD of 11.7 ± 11.8 h before onset of AKI. Similar findings were noted when specificity was increased by using a cut-off uNGAL value of 185 ng/mL.

## 4. Discussion

To our knowledge, this is the largest study that explores the association between uNGAL and AKI in neonates (<28 days) undergoing cardiac surgery with CPB. We found a significant association between elevated post-operative uNGAL and AKI between 4 and 16 h post-operatively. Although the elevation of uNGAL >100 ng/mL occurred 8.7 or more hours earlier than the onset of AKI in neonates with AKI, a similar but transient early uNGAL elevation occurred in neonates without AKI (Figure 2). Thus, in contrast to findings in older children and in spite of the association between uNGAL and AKI in neonates, an early rise in post-operative uNGAL was not a useful early predictor of AKI for neonates in our study. 

AKI occurred in 22% (17 of 76) of our neonatal patients. This is a lower incidence of AKI than frequently cited [9,22,23] but consistent with our internal prevalence of AKI in neonates undergoing CPB, with the largest previously reported subgroup of neonates exposed to CPB, and with the largest multi-centered study looking at AKI exclusively in neonates [6,24]. No neonates had preoperative AKI in our study. In contrast to prior work, in our study AKI occurred more frequently in neonates who were older at time of surgery and was unrelated to duration of bypass and complexity of cardiac surgery (RACHS score). Reasons for this association are likely complex. First, our inclusion criteria selected for the most critically ill neonates who required early cardiac repair, so that all neonates in our study were young, had complex congenital heart disease, and were exposed to prolonged CPB times (>120 min). Additionally, prior work has demonstrated that later surgical repair (or increased age at time of cardiac surgery) in neonates with congenital heart disease is associated with decreased hemoglobin saturation and increasing white matter injury [25,26]. Neonatal AKI occurrence may be similarly related to worsening oxygen delivery when surgical repair is delayed in these critically ill neonates. 

Prior to this study, research investigating the relationship between uNGAL and AKI in neonates undergoing cardiac repair using CPB has been limited. In contrast, a robust pediatric literature, examining more than 2200 children, describes uNGAL’s association with and ability to predict AKI in older children [11,13,18,19,24,27,28,29,30,31,32]. In general, these studies demonstrate an abrupt early rise in uNGAL post-operatively from low preoperative baseline values in those children who develop AKI. In those who do not develop AKI, uNGAL values remain at or near their low preoperative value. The early rise in uNGAL among those who develop AKI precedes by hours to days of a corresponding rise in sCr that justifies the diagnosis of AKI; thus, the early rise in uNGAL is termed “predictive” of AKI. 

We did not observe this typical pattern. Our neonates showed abrupt early post-operative rises in uNGAL regardless of AKI status. Only between 4 and 16 h post-CPB did we find a widening separation in uNGAL values that accounts for the association between elevated uNGAL and AKI, shown in Figure 2. These plots also demonstrate the wide variability in post-operative uNGAL, accounting for its limited ability to discriminate AKI from non-AKI regardless of uNGAL cut-off values used. 

Several other studies have shown results that deviated from the typical pediatric post-operative pattern of uNGAL; and these studies are predominantly in younger populations. In the TRIBE consortium, for example, the strength of the association between elevated uNGAL and AKI was diminished in patients <2 years of age [29]. Among infants <1 year of age, both Adams et al. and Ruff et al. demonstrated no difference in post-CPB uNGAL values between those with and without AKI [27,28]. In a smaller subgroup of neonates undergoing CPB, Krawczeski et al. also noted post-CPB rises from baseline in uNGAL regardless of AKI status in the 35 neonates studied; however, in the eight neonates who developed AKI, the early uNGAL rise was steep enough to provide discrimination between AKI and no-AKI at 2 h post-CPB [19]. It is notable, that Krawczeski et al. found optimal sensitivity and specificity for uNGAL in their neonates occurred at a value of 185 ng/mL. In our cohort, using this higher cut off yielded increased specificity but decreased sensitivity in AKI discrimination compared to the lower cut off of 100 ng/mL. Defining the optimal risk balance between over-diagnosis (increased sensitivity at the expense of specificity) and under-diagnosis (the reverse) in a particular group of patients remains a matter of clinical judgment. 

What leads to the limited ability of uNGAL to discriminate between AKI and non-AKI states in neonates? We found wide variations among neonates in both sCr and uNGAL pre- and post-operatively. Preoperative elevations in uNGAL >50 ng/mL occurred in many neonates without evidence of preoperative AKI defined by KDIGO criteria; and preoperative (baseline) uNGAL elevations were evenly distributed between neonates who did and did not develop AKI post-operatively. This is in contrast to the findings in older children where elevated uNGAL correlated well with severe AKI, need for RRT, and increased mortality [24,33]. This wide range of baseline uNGAL in neonates may reflect variable maturity in tubular function or nephron number consistent with prior work which demonstrated that reference ranges for uNGAL change with both gestational and postnatal age [34,35,36,37]. Additionally, increased baseline uNGAL variability may reflect differential alterations in both renal perfusion and oxygenation associated with diverse congenital cardiac lesions [13,24,38]. 

Also notable is our finding that sCr was frequently lower post-operatively than preoperatively (45%, 34 of 76 patients). AKI was equally common in those with lower and those with equal or higher post- than preoperative sCr. The explanation for these findings is again likely complex. The normal descent of sCr values from maternal baseline values at birth to much lower equilibration levels occurs over a period of about 2–3 weeks in term neonates, resulting in a fall of approximately 0.04 mg/dL/d [39]. In our study, this rate of fall was doubled (0.08 mg/dL/d) in those neonates with lower post-operative sCr, making it unlikely that normal descent, if it even occurs in the circumstances of cardiac surgery, fully explains the difference. A second consideration is CPB itself. Depending on the priming volume of the bypass circuit used, a neonate’s volume of distribution may be increased 8–40% through the use of CPB and thus the decrease in sCr may be dilutional. Moreover, post-operative fluid shifts and the need for intravascular volume repletion are common and might impact sCr measurement [40,41]. 

These multiple influences on post-operative sCr decrease its reliability as a marker of renal function and increase the uncertainty of AKI diagnosis, which is already problematic in neonates [3,4,5,11,12,21]. Both the incidence of AKI and the strength of the association between uNGAL and AKI are likely underestimated. Recent work by Askenazi et al. suggests that substantial decreases in threshold for absolute sCr rise from current KDIGO criteria may be required to optimally capture neonates with AKI in the first postnatal week [42]. Using Askenazi et al.’s criteria of sCr rise of 0.1 mg/dL would have yielded a diagnosis of AKI in an additional 24% (18 of 76) of neonates in our cohort. In neonates, further work is needed both to determine how best to define AKI in the first postnatal weeks as the current “gold standard” for AKI remains flawed in this population. 

### Strengths and Limitations

Strengths of our study are that this is a prospective observational study among a relatively homogenous group of neonates with critical congenital heart disease. The number of true neonates (<28 days of age) requiring CPB is small. We were able to take advantage of our institution’s Neonatal Cardiac Intensive Care Unit, which serves as an international referral center, to screen 237 patients and enroll 76 patients for this study. Thus while our study is relatively small, it remains the largest study of uNGAL in neonates <28 days of age undergoing cardiac surgery to date. Given the unreliability and variation of sCr in the neonatal population, a critical limitation in our study is the use of a sCr-based definition of AKI as the gold standard for AKI. Further limitations of this study are that this was a single-center study, only a single biomarker was analyzed, and sample size was not large enough to stratify data according to cardiac lesion or AKI stage. We recognize that the physiologic (and ischemic) implications of different congenital heart diseases may be critically important not only to the risk posed for injury to the kidney, but also may have significant implications as to the underlying renal function [11,13,18,24,27,29]. In children, uNGAL’s ability to predict AKI is enhanced when it is used in combination with other biomarkers for renal injury or function such as interleukin-18 (IL-18), Kidney Injury Molecule-1 (KIM-1), and tissue inhibitor of metalloproteinases 1 (TIMP-1) [29,31]. We examined only the association of uNGAL and AKI, as this study was part of a series of studies examining the utility of uNGAL as a biomarker for various outcomes [19,22,43,44]; however, further investigation of the utility of multiple biomarkers in this population is warranted. Finally, while to the best of our knowledge neonates included in our study had normal renal anatomy and function, there may be microscopic changes that could not be appreciated by ultrasound. 

## 5. Conclusions

Elevated uNGAL is associated with AKI in neonates between 4 and 16 h post-operatively. However, this relationship is more complex than that seen in older children. Wide variability of uNGAL (both at baseline and post-operatively) and of sCr post-operatively is likely related to the interplay between normal age-related alterations in renal function and the renal effects of the cardiac lesion, CPB, and surgery. These factors compromise the utility of both uNGAL as a marker for neonatal renal injury as well as the definition of neonatal AKI based solely on changes in sCr and urine output. These findings emphasize the need to develop reliable biomarkers and definitions of renal injury in vulnerable populations with complex physiology.

## Figures and Tables

**Table 1:** Characteristics of study sample by AKI status.

	AKI *n* = 17	No AKI *n* = 59	Total *n* = 76	*p*-Value
**Categorical variables, *n* (%)**				
Male sex	12 (71)	37 (63)	53 (70)	0.55
Elevated baseline uNGAL >50 ng/mL	4 (33)	15 (33)	19 (33)	0.99
Elevated baseline uNGAL >100 ng/mL	2 (25)	12 (26)	15 (26)	0.99
Lower post-operative sCr	5 (29)	29 (49)	34 (45)	0.15
RACHS-1 score				0.83
Scores 2–3	4 (24)	17 (29)	21 (28)	
Score 4	8 (47)	22 (37)	30 (39)	
Scores 5–6	5 (29)	20 (34)	25 (33)	
**Continuous variables, median [IQR]**				
Birthweight (kg)	3.03 [2.87–3.23]	3.23 [2.97–3.59]	3.19 [2.94–3.54]	0.15
Gestational age (wk)	38 [37–39]	39 [38, 39]	39 [38, 39]	0.15
Age at surgery (d)	7 [6–11]	5 [4–7]	6 [4–8]	0.01
Baseline sCr (mg/dL)	0.46 [0.37–0.60]	0.50 [0.40–0.64]	0.50 [0.38–0.64]	0.51
Baseline uNGAL (ng/mL)	19 [8–90]	22 [8–98]	22 [8–98]	0.64
Peak uNGAL (ng/mL)	763 [454–1256]	319 [119–807]	436 [125–845]	0.03
Bypass time (min)	137 [114–173]	156 [120.5–182]	155 [120–182]	0.51
Cross-clamp time (min)	76 [56.5–100.5]	83 [66–101]	82 [63–102]	0.52
ACP time (min)	53 [47.75–58.25]	55 [30–57.5]	55 [32–58]	0.76
Circulatory arrest time (min)	29 [12–46.5]	24 [8–36]	25 [8–38.25]	0.51

IQR = interquartile range. RACHS-1 = Risk Adjustment for Congenital Heart Surgery. ACP = antegrade cerebral perfusion. Kg = kilogram. Wk = week. D = day. Min = minutes.

**Table 2:** Characteristics of AKI status.

	KDIGO Criteria		
KDIGO Staging	Increased Creatinine	Oliguria	Total (*n* = 17)
Stage 1	9	3	9
Stage 2	6	3	6
Stage 3	2	2	2

Stage 1—increase in sCr (≥0.3 mg/dL or 1.5–1.9 x rise from previous lowest value) or decreased urine output (<1 mL/kg/h for 24 h). Stage 2—increase in sCr (≥2–2.9 x rise from previous lowest value) or decreased urine output (<0.3 mL/kg/hr for 24 h). Stage 3—increase in sCr (≥3 x rise from previous lowest value) or decreased urine output (<0.5 mL/kg/h for 24 h).

**Table 3:** Median [IQR] uNGAL values at urine collection intervals for neonates with and without AKI. Odds ratios for AKI versus No AKI derived logistic regression models at each uNGAL collection interval.

	Median uNGAL [IQR ^a^] (ng/mL)			Logistic Regression Models ^b^	
Time (h)	AKI	No AKI	*p*-Value ^c^	OR (95% CI)	*p*-Value
0	19 [8, 90]	22 [8, 90]	0.64	1.02 (0.65, 1.59)	0.93
3	70 [28, 662]	226 [72, 468]	0.35	0.86 (0.57, 1.34)	0.49
4	230 [24, 459]	149 [44, 444]	0.89	1.05 (0.71, 1.60)	0.79
7	361 [106, 838]	76 [25, 196]	0.01	2.22 (1.34, 4.08)	0.004
10	405 [107, 773]	63 [24, 185]	0.003	3.48 (1.58, 8.71)	0.002
13	259 [41, 570]	54 [23, 158]	0.02	2.15 (1.25, 4.10)	0.01
16	61 [42, 470]	40 [18, 72]	0.03	1.95 (1.14, 3.60)	0.02

^a^ Interquartile range (IQR). ^b^ Logistic models regress AKI on log(uNGAL), controlling for gestational age, sex, CBP time, RACHS score, and chronological age at surgery. ^c^ Obtained using Mann–Whitney test.
